# Progressive high-load strength training compared with general low-load exercises in patients with rotator cuff tendinopathy: study protocol for a randomised controlled trial

**DOI:** 10.1186/s13063-014-0544-6

**Published:** 2015-01-27

**Authors:** Kim G Ingwersen, Robin Christensen, Lilli Sørensen, Hans RI Jørgensen, Steen Lund Jensen, Sten Rasmussen, Karen Søgaard, Birgit Juul-Kristensen

**Affiliations:** Institute of Sports Science and Clinical Biomechanics, University of Southern Denmark, Campusvej 55, DK-5230 Odense, Denmark; Physiotherapy Department, Hospital Lillebaelt, Vejle Hospital, Kabbeltoft 25, DK-7100 Vejle, Denmark; Department of Rheumatology, Musculoskeletal Statistics Unit, The Parker Institute, Copenhagen University Hospital, Bispebjerg and Frederiksberg, Nordre Fasanvej 57, road 8, entrance 19, DK-2000 Frederiksberg, Denmark; Orthopaedic Department, Shoulder Unit, Hospital Lillebaelt, Vejle Hospital, Kabbeltoft 25, DK-7100 Vejle, Denmark; Orthopaedic Department, Shoulder Unit, Odense University Hospital, Svendborg Hospital, Valdemarsgade 53, DK-5700 Svendborg, Denmark; Department of Orthopaedic Surgery, Shoulder Unit, Aalborg University Hospital, Farsø Hospital, Højgårdsvej 11, DK-9640 Farsø, Denmark; Orthopaedic Surgery Research Unit, Aalborg University Hospital, Sdr. Skovvej 15, DK-9000 Aalborg, Denmark; Department of Clinical Medicine, Aalborg University, Sdr. Skovvej 15, DK-9000 Aalborg, Denmark; Department of Health Sciences, Institute of Occupational Therapy, Physiotherapy and Radiography, Bergen University College, Inndalsveien 28, NO-5063 Bergen, Norway

**Keywords:** Shoulder, Impingement, Tendinopathy, Exercise, Rotator cuff

## Abstract

**Background:**

Shoulder pain is the third most common musculoskeletal disorder, often affecting people’s daily living and work capacity. The most common shoulder disorder is the subacromial impingement syndrome (SIS) which, among other pathophysiological changes, is often characterised by rotator cuff tendinopathy. Exercise is often considered the primary treatment option for rotator cuff tendinopathy, but there is no consensus on which exercise strategy is the most effective. As eccentric and high-load strength training have been shown to have a positive effect on patella and Achilles tendinopathy, the aim of this trial is to compare the efficacy of progressive high-load exercises with traditional low-load exercises in patients with rotator cuff tendinopathy.

**Methods/Design:**

The current study is a randomised, participant- and assessor-blinded, controlled multicentre trial. A total of 260 patients with rotator cuff tendinopathy will be recruited from three outpatient shoulder departments in Denmark, and randomised to either 12 weeks of progressive high-load strength training or to general low-load exercises. Patients will receive six individually guided exercise sessions with a physiotherapist and perform home-based exercises three times a week. The primary outcome measure will be change from baseline to 12 weeks in the patient-reported outcome Disabilities of the Arm, Shoulder and Hand (DASH) questionnaire.

**Discussion:**

Previous studies of exercise treatment for SIS have not differentiated between subgroups of SIS and have often had methodological flaws, making it difficult to specifically design target treatment for patients diagnosed with SIS. Therefore, it was considered important to focus on a subgroup such as tendinopathy, with a specific tailored intervention strategy based on evidence from other regions of the body, and to clearly describe the intervention in a methodologically strong study.

**Trial registration:**

The trial was registered with Clinicaltrials.gov (NCT01984203) on 31 October 2013.

## Background

People with pain in the neck and shoulder region are often disabled to the point where they cannot live a normal life. The pain may also influence the person’s work capacity and financial as well as social situation. Pain in the shoulder is the third most common musculoskeletal disorder, and the lifetime prevalence is estimated to be between seven and 10% [1]. The prevalence for daily non-specific shoulder pain in a normal working population in an industrialised country is estimated to be 12% [2]. Furthermore, up to 23% of the workforce seeking care from the healthcare system with a shoulder problem have been sick-listed for more than one week [3].

Clinically, the most common shoulder-related cause of contact with the healthcare system is subacromial impingement syndrome (SIS), which accounts for 33% of all shoulder-related healthcare contact [4,5]. SIS is generally considered to be a cluster of symptoms, rather than a single pathology. The Complaints of the Arm, Neck and/or Shoulder (CANS) model defines SIS as ‘Disorders that include the rotator cuff syndrome, tendonitis of the m. infraspinatus, m. supraspinatus and m. subscapularis, and bursitis in the shoulder area’ [6]. The cause of SIS is considered to be multi-factorial and involves an altered muscle recruitment pattern in the rotator cuff and thoracoscapular muscles, potentially in combination with alterations to the anatomical structures in the subacromial space. These alterations may result in varying degrees of micro-traumas and degenerative pathophysiological changes in the rotator cuff muscle and surrounding tissue, which eventually can result in rotator cuff tendinopathy. Generally, the clinically relevant pathophysiological conditions associated with the diagnosis of SIS, such as rotator cuff tendinopathy, are often difficult to identify objectively [7,8], and consequently the diagnosis of rotator cuff tendinopathy is primarily based on a combination of the patient’s history, clinical tests and ultrasonographic visualisation of changes in the rotator cuff tendons.

The Danish national clinical guidelines for the treatment of SIS recommend active rehabilitation strategies, and in cases where three to six months of exercise has been ineffective, arthroscopic subacromial decompression surgery can be considered [9]. These recommendations are, amongst others, based on the evidence that exercise has shown results comparable to arthroscopic subacromial decompression when evaluated on pain and patient-reported function [10-14]. However, the recommended guidelines rarely describe specific exercise programmes, nor are they sufficient in detail to be targeted at subgroups of SIS, such as rotator cuff tendinopathy. This means there is no consensus on specific rehabilitation exercise programmes for SIS patients with rotator cuff tendinopathy.

Among patients with Achilles or patella tendinopathy, studies of eccentric or progressive high-load exercises have shown promising effects on pain and function [15], and such exercises for chronic tendinopathy are often considered to be the first line of treatment [16-18]. Some of the mechanisms believed to be responsible for the positive outcomes of eccentric exercises are reduced structural abnormalities, such as hypoechoic areas, irregular structures, neovascularisation and tendon thickness [19,20]. The reasons for these changes could possibly be increased collagen type I synthesis [21]. Considering that collagen synthesis peaks at between 24 and 72 hours post-exercise [22], programmes prescribing daily training, such as those recommended for standard eccentric exercise programs, might not be optimal [23]. In support of this, very high-load eccentric training with relatively few repetitions and low frequency (twice a week) has shown equally beneficial effects on patella tendinopathy [24,25]. Also, concentric heavy slow resistance exercises, without emphasis on the eccentric component, three times a week [25] has shown positive effects on patella tendinopathy. Furthermore, heavy slow resistance exercises seem to reduce abnormal fibrillar morphology among patients with patella tendinopathy [26].

However, rotator cuff tendinopathy is often treated as any other subgroup of SIS, with a combination of low-load exercises for internal and external rotation of the shoulder joint, with an attempt to target the rotator cuff muscles and optimise muscle coordination. Only a few studies have used progressive exercises or eccentric exercises in patients with SIS, and they have reported positive results on pain and function [27-32]. But all these studies have primarily targeted SIS and did not differentiate between the underlying pathologies. Inclusion of different subgroups of SIS in the same trial makes it difficult to customise an optimal exercise programme and, possibly, to detect a clinically relevant change between different exercise strategies [8].

Based on the evidence reported from studies on knee, Achilles and elbow tendons, applying eccentric or high-load exercise programmes in patients with rotator cuff tendinopathy could be beneficial for patients’ pain, function and quality of life [8], and for ultrasonographic visualised structural changes [33-35], when compared with traditional exercise strategies. But the rationale for using progressive high-load exercises is the diagnosis of tendinopathy and therefore, unlike existing studies, a study of eccentric or progressive high-load exercises should include only patients with tendinopathy. To our knowledge, no studies have investigated the effect on pain and function of progressive high-load exercises compared with traditional low-load exercises in the subgroup of SIS patients primarily showing signs of rotator cuff tendinopathy. A proportion of patients diagnosed with tendinopathy will also have a concomitant pathology in the surrounding tissue of the glenohumeral joint. Often corticosteroid injection into the subacromial space is used as an adjunct treatment for exercise interventions, if regarded relevant by the orthopaedic specialist. It is uncertain if this concomitant treatment has an additional effect on the exercise treatment.

The aim of the study is to determine in a randomised, double-blind controlled trial if a progressive high-load exercise programme for the rotator cuff muscles is superior to a traditional low-load exercise programme in patients with rotator cuff tendinopathy, based on the primary endpoint; change in score on the Disabilities of the Arm, Shoulder and Hand (DASH) questionnaire [36] after 12 weeks of exercise. The second aim is to evaluate the possible interaction of concomitant use of corticosteroids with the exercise intervention.

## Methods/Design

### Study design

**Figure 1:**
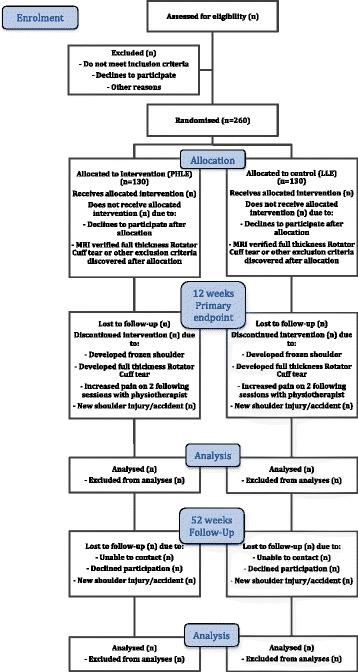
**Expected flow of participants through the study.** LLE: low-load exercises; PHLE: Progressive high-load exercises.

This trial, called the Rotator Cuff Tendinopathy Exercise (RoCTEx) trial, is a multicentre (three sites), stratified (by corticosteroid injection (yes or no)), randomised, controlled, observer- and patient-blinded superiority trial, with a two-group parallel design, to be conducted in Denmark. The primary endpoint will be 12 weeks after baseline. Patients will be randomised to either progressive high-load exercises or low-load exercises (block randomisation with a 1:1 allocation). As illustrated in Figure 1, patients will also be assessed at one year from the baseline, referred to as the follow-up assessment.

### Settings and locations

Patients will be recruited from the shoulder units at the outpatient clinics of orthopaedic departments at the Hospital Lillebaelt - Vejle Hospital, Aalborg University Hospital - Farsø Hospital and Odense University Hospital - Svendborg Hospital.

### Participants

The orthopaedic specialists will perform a standardized initial screening of all patients referred to the shoulder units. If patients fulfil the eligibility criteria they will be referred to the principal investigator, who will perform the final eligibility assessment and give detailed information about the study. Patients will be invited to participate if they meet the following inclusion criteria: between 18 and 65 years of age, have a current shoulder complaint lasting at least three months prior to the time of enrolment, pain is located in the proximal lateral aspect of the upper arm (C5 dermatome) that is aggravated by abduction, a positive ‘Full Can Test’ and/or Jobe’s test and/or Resisted External Rotation Test, a positive Hawkins-Kennedy test and/or Neer’s test, ultrasonographic verification of either tendon swelling, and/or presence of hypoechoic areas, calcification, fibrillar disruption and/or neovascularisation in the m. supraspinatus.

Patients will be excluded if they fulfil any of the following exclusion criteria: Resting pain more than 40 mm on a visual analogue pain scale (range: 0 to 100), bilateral shoulder pain, less than 90 degrees of active elevation of the arm, a full thickness rupture in the rotator cuff tendon verified by ultrasonography, presence of calcification larger than 5 mm (vertical distance) verified by x-ray, corticosteroid injection within the last six weeks, a radiologically verified fracture, glenohumeral osteoarthritis, prior surgery or dislocation of the affected shoulder, a clinically suspected labrum lesion, symptomatic arthritis in the acromioclavicular joint, a frozen shoulder or symptoms derived from the cervical spine, sensory or motor deficit in the neck or arm, suspected competing diagnoses (for example, rheumatoid arthritis, cancer, neurological disorders, fibromyalgia, psychiatric illness), pregnancy or inability to understand spoken and written Danish.

### Interventions

Generally, both groups will receive the same exercises (although they differ on the amount of load), attention and basic information about ergonomic corrections related to the daily use of the upper extremity. The exercise program will consist of six exercises: two exercises for the scapula stabilising muscles (push-up plus exercise and low row exercise), two for the rotator cuff muscles (scaption and side-lying external rotation), and two stretching and mobility exercises (posterior capsule stretch and scapulae retraction). Patients will be instructed in performing home-based training three times per week, and will be seen for initial instruction in week one (60 minutes), and follow-up exercise sessions (30 minutes) in weeks two, three, four, six and nine. The general information about the project and exercise instructions will be given through a DVD to minimise the influence of the physiotherapist. On the first visit to the physiotherapist, the patients will receive detailed instructions on how to perform the exercise programme based on the instructions on the DVD. At the follow-up exercise sessions corrections will be made, and weights adjusted. The patients will be given a copy of the DVD and an exercise brochure to enable them to follow the instructions and exercises at home.

The difference between the intervention and control groups will be the external load targeting exercises for the rotator cuff. The intervention group will perform 12 weeks of progressive high-load exercises, gradually increasing the load from 15 repetitions maximum (RM) in week one to six RM in weeks nine to 12. The intervention group will only be allowed to do isometric exercises if the pain exceeds 50 mm on the visual analogue scale. The control group will perform 12 weeks of low-load exercises, performed with 20 RM from weeks one to 12. For both groups, scapulae exercises will be performed with 20 RM, and stretching and mobility exercises will be performed five times, for 20 seconds each, in weeks one to 12.

Concomitant use of corticosteroid injections will be based on the orthopaedic specialist’s evaluation, in accordance with the department’s standard procedures. Between 25 and 50% of patients are expected to receive injections. The corticosteroid injections will be given to patients at the shoulder unit of the orthopaedic departments, after baseline assessment but before randomisation. Randomisation will be performed in a stratified manner to ensure even distribution of patients receiving and not receiving corticosteroid injections in the two exercise groups.

### Outcome measures

The primary outcome measure will be change from baseline to week 12 in the patient-reported outcome Disabilities of the Arm, Shoulder and Hand (DASH) questionnaire (0 to 100 score, 100 = no problems) [36], and reported as the difference in mean change between the groups. The secondary endpoint assessment on the primary outcome will be performed 12 months after baseline (Figure 1).

The secondary outcome, measured at baseline and after 12 weeks, will include: the Shoulder injury and Osteoarthritis Outcome Score (SOOS; 0 to 100 score, 100 = no problems) [37], the Hospital Anxiety and Depression Scale (HAD Scale; 0 to 21 score, 0 = no problems) [38], EuroQol instrument (EQ-5D index; −0.59 to 1 score, −0.59 = lowest health-related quality of life), EuroQol Visual Analogue Scale (EQ-VAS; 0 to 100 score, 0 = lowest health-related quality of life) [39], perceived pain at rest, general activity and maximum pain experienced during the previous 24 hours (visual analogue scale, 0 to 100 score, 0 = no pain) [40], maximum isometric voluntary contraction (MVC) of external and/or internal rotation and scaption measured by dynamometers, active and passive range of movement in scaption (0 to 180 degrees), external (0 to 90 degrees) and internal rotation (0 to 90 degrees) measured by goniometer, clinical tests (Scapula Assistance Test [41], Scapula Retraction Test (positive or negative) [42] and ultrasonography measurements (aggregated sum of: supraspinatus tendon thickening (grade 0 to 3), fibrillar disruption (grade 0 to 3), calcification (grade 0 to 3) and neovascularisation (grade 0 to 4) [33]).

The following secondary outcomes will only be measured at the 12-month follow-up assessment: number of patients referred for, or completed, arthroscopic subacromial decompression; patients given subacromial corticosteroid injections after the intervention period; the number of visits to a general practitioner or secondary healthcare setting due to the shoulder problem (registration in the Danish Central Office of Patient Registration); and economic variables (for example, patient-reported number of sick days from work and sport attributed to the shoulder and time spent on rehabilitation in the trial period and post-trial, (patient-reported and register-controlled). Table 1 presents all outcome measures and at which time point they will be collected.

**Table 1:** **Summary of measures to be collected**

**Article number**	**Variable**	**Baseline**	**Week 3**	**Week 6**	**Week 9**	**Week 12**	**Week 52**
Socio-demographic measurements							
1, 2, 3	Age - year	Yes	n/a	n/a	n/a	n/a	n/a
1, 2, 3	Female sex – number (%)	Yes	n/a	n/a	n/a	n/a	n/a
1, 2, 3	Duration of symptoms - year	Yes	n/a	n/a	n/a	n/a	n/a
	Symptom history						
1, 2, 3	- Accident or acute incidence	Yes	n/a	n/a	n/a	n/a	n/a
1, 2, 3	- Slow consistent development (overload)	Yes	n/a	n/a	n/a	n/a	n/a
1, 2, 3	- Fluctuating development	Yes	n/a	n/a	n/a	n/a	n/a
	Compliance with exercise protocol						
1, 2, 3	- Visits to the physiotherapy department	n/a	n/a	n/a	n/a	Yes	n/a
1, 2, 3	- Exercise diary	n/a	n/a	n/a	n/a	Yes	n/a
1, 2, 3	- Pain diary	n/a	n/a	n/a	n/a	Yes	n/a
Patient reported and physiological measurements							
1, 2, 3	Disabilities of the Arm, Shoulder and Hand (DASH) questionnaire (range: 0 to 100) [36]	Yes	Yes	Yes	Yes	Yes	Yes
1,2	Shoulder injury and Osteoarthritis Outcome Score (SOOS) [37]	Yes	n/a	n/a	n/a	Yes	Yes
1, 2	Hospital Anxiety and Depression Scale (HAD Scale, range: 0 to 21) [38]	Yes	n/a	n/a	n/a	n/a	n/a
1, 2	Visual analogue pain scale (VAS, range: 0 to 100) [40]						
	- Rest	Yes	n/a	n/a	n/a	Yes	n/a
	- Maximum	Yes	n/a	n/a	n/a	Yes	n/a
	- Activity	Yes	n/a	n/a	n/a	Yes	n/a
	Isometric strength						
1, 3	- Scaption	Yes	n/a	n/a	n/a	Yes	n/a
1, 3	- External rotation	Yes	n/a	n/a	n/a	Yes	n/a
1, 3	- Internal rotation	Yes	n/a	n/a	n/a	Yes	n/a
	Degree of active movement						
1, 3	- Scaption	Yes	n/a	n/a	n/a	Yes	n/a
1, 3	- External rotation	Yes	n/a	n/a	n/a	Yes	n/a
1, 3	- Internal rotation	Yes	n/a	n/a	n/a	Yes	n/a
	Positive clinical test – number (%)						
3	- SAT [41]	Yes	n/a	n/a	n/a	Yes	n/a
3	- SRT [42]	Yes	n/a	n/a	n/a	Yes	n/a
	Ultrasonographic measurement [33]						
1	- Hypoechoic scale (range: 0 to 3)	Yes	n/a	n/a	n/a	Yes	n/a
1	- Neovascularity scale (range: 0 to 4)	Yes	n/a	n/a	n/a	Yes	n/a
1	- Tendon swelling – mm	Yes	n/a	n/a	n/a	Yes	n/a
1	- Calcification (range: 0 to 3)	Yes	n/a	n/a	n/a	Yes	n/a
Socio-economic measurements							
	Referred to						
1, 2	- Operation – number (%)	n/a	n/a	n/a	n/a	Yes	Yes
1, 2	- Corticosteroid injection(s) – number	Yes	n/a	n/a	n/a	Yes	Yes
	Shoulder related						
2	- Visits to general practitioner – number	n/a	n/a	n/a	n/a	Yes	Yes
2	- Visits to secondary healthcare setting – number	n/a	n/a	n/a	n/a	Yes	Yes
2	- Sick days – number	n/a	n/a	n/a	n/a	Yes	Yes
2	Time spent on shoulder-related rehabilitation - hours	n/a	n/a	n/a	n/a	Yes	Yes
2	EuroQol-5D (EQ-5D, range: 0 to 100) [39]	Yes	n/a	n/a	n/a	Yes	Yes

n/a: not assessed; SAT: Scapulae Assistance Test; SRT: Scapulae Retraction Test.

Article 1: Progressive high-load exercise compared with general low-load exercise in patients with rotator cuff tendinopathy: a randomised trial.

Article 2: The effect of 12 weeks of progressive high-load exercise in patients with rotator cuff tendinopathy on patient-reported and economic outcomes – 12 months follow-up.

Article 3: The prognostic evaluation of clinical tests in patients with rotator cuff tendinopathy.

Demographic data and other measurements will be recorded, covering socio-economic variables such as: employment, education, statement of income, history of injury (for example, accident, slow development and fluctuations), duration of current symptoms and physical demands in work and/or hobby activities (patient-reported).

The patients will be instructed to complete an exercise and pain diary. In the exercise diary, the patients will be asked to report the number of sets and repetitions per set for every training session, as well as the load during the exercise. Self-reported pain will be registered immediately before and 30 minutes after each training session. All pain measurements will be reported on the Numeric Pain Rating Scale. In addition to pain, patients will be asked to report use of analgesics (type and amount).

Good compliance will be defined by attendance at four out of the six visits to the physiotherapist, and an 80% complete exercise diary, where 100% will be defined by performance of 36 sessions of training (12 weeks at three training sessions per week).

### Data collection

Three outcome assessors will perform all enrolment, baseline and follow-up assessments. Before starting the data collection, the assessors will train together with the primary investigator and decide on a consensus standard of interpreting all outcome variables.

### Sample size and power considerations

**Figure 2:**
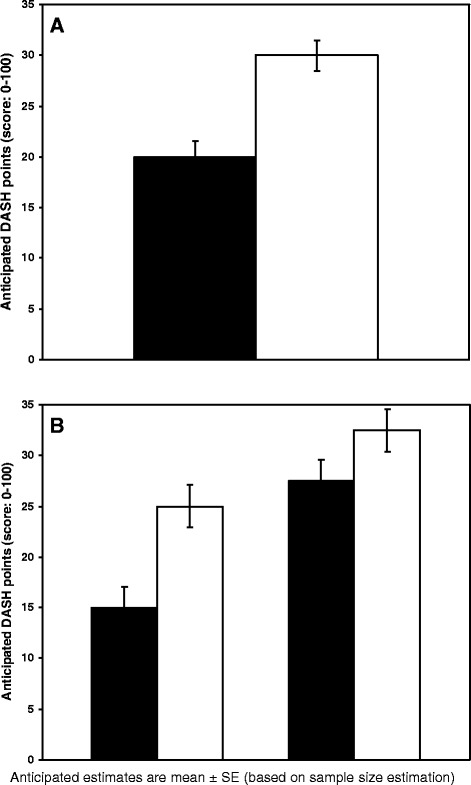
**Anticipated outcome (primary and secondary objectives). A**: (Primary objective) Illustration of expected DASH score at 12 weeks. Black = intervention group, White = control group. **B**: (Secondary objective) Illustration of expected DASH score at 12 weeks stratified for concomitant corticosteroid injection. Columns I + II = intervention group, Columns III + IV = control group, Black = concomitant corticosteroid injection, White = no concomitant corticosteroid injection. DASH, Disabilities of the Arm, Shoulder and Hand Questionnaire.

Studies on patients with SIS have shown mean baseline values for DASH scores between 18 and 65, and standard deviations (SD) between 12 and 20 [30,31,43-47]. Often a 50% reduction in DASH score is seen after treatment [30,31,44,46,47], and it is suggested that a 40% change corresponds to a substantial improvement for the patient [46]. Based on these data, we expect a mean baseline of 40 points on the DASH questionnaire with an SD of 17. We expect a 50% change (change from 40 to 20 DASH points) in the progressive high-load exercise group, and 25% (change from 40 to 30 DASH points) in the low-load exercise group (Figure 2A).

For a two-sample pooled t-test of a normal mean difference with a two-sided significance level of 0.05 (*P* ≤0.05), assuming a common SD of 17 DASH points, a sample size of 100 patients per group has a power of 0.985 (>95% power for the primary outcome) to detect a between-group mean difference of 20 and 30 DASH points after 12 weeks in the progressive high-load exercise group and the low-load exercise group, respectively.

As the second objective is to explore the interaction between corticosteroid use (yes or no) and exercise (progressive high-load exercise or low-load exercise), it is hypothesised that, in the high-load group receiving corticosteroids at baseline, the DASH score at endpoint (12 weeks) will be more favourable than those without corticosteroids. With an SD of 17 DASH points, a sample size of 62 patients per stratum (having progressive high-load exercise) will be required to obtain a power of at least 0.9 (90%) to detect a mean difference of 10 DASH points between the two subgroups within the progressive high-load exercise group (Figure 2B). Consequently, it is decided to include 260 patients in the study, assuming a similar number of patients injected and not injected with corticosteroids (1:1; 2 × 130 patients randomly assigned to each exercise group (progressive high-load exercise versus low-load exercise)).

A final deadline for patient recruitment is set at Marts 2015, meaning that patient recruitment stops when the total number of 260 patients has been recruited, or the deadline of Marts 2015 is reached. In case the target number of 260 patients has not been met within the specified recruitment period, the power of the study will be less than expected. With an SD of 17 DASH points, a sample size of 46 patients per group will be required to obtain a power of at least 0.8 (80%) to detect a mean difference of 10 DASH points between the two groups.

### Randomisation and allocation concealment

Patients will be randomly assigned to either of the two exercise groups with a 1:1 allocation as per a computer-generated randomisation schedule, stratified by administration of concomitant corticosteroid injection using permuted blocks of random sizes (two to six). The primary investigator, assessors and administrator of the randomisation procedure will not know block sizes in order to ensure allocation concealment. In practice, after recruitment and baseline measurements, the secretary at the physiotherapy department will administer the allocation procedure by taking a sequentially numbered, opaque sealed envelope from one of two ring binders, determined by the potential referral to corticosteroids (yes or no) by the orthopaedic specialist.

### Blinding

Outcome assessors will perform both baseline and follow-up assessments, and will be kept blinded from treatment allocation. At the follow-up assessments, patients will be strongly encouraged not to disclose the components of their exercise programme, in order to keep the outcome assessor blinded. Patients will be blinded regarding exercise allocation by informing them that the trial involves testing two exercise programmes consisting of the same exercises, and that they will be performed in two different ways, but not defining the specific difference between these, or which is hypothesised to be more effective.

### Statistical analysis plan

The primary efficacy analysis performed is assessment of the between-group difference in change in the DASH score after 12 weeks in the intent-to-treat (ITT) population (all randomised patients independent of compliance and withdrawals). In the case of missing data due to drop-outs, a non-responder imputation will be applied; a baseline observation carried forward (BOCF) technique will be used for patients who do not complete the study (as will be illustrated in a corresponding trial flow diagram).

For the primary analyses at week 12, we will use analysis of covariance (ANCOVA) to compare the progressive high-load exercise group with the low-load exercise group for mean changes from baseline in the DASH score, as well as the secondary continuous outcomes. The primary model includes the change from baseline as the dependent variable, with treatment group (progressive high-load exercise or low-load exercise), corticosteroid status (yes or no), and the centre site (one, two or three) as main effects, with the baseline score as an additional covariate. The secondary analysis for the primary outcome will add the interaction term for group × corticosteroid in the primary model.

To analyse the longitudinal element of time effects of DASH in the randomised trial (repeated measures design three, six, nine, and 12 weeks), a linear approach will be used, fitted in SAS software (version 9.3 Service Pack 4; SAS Institute Inc., Cary, North Carolina, United States) using the procedure ‘PROC MIXED’ based on restricted maximum likelihood (REML) estimates of the parameters. The variable ‘patient’ will be applied as a random effects factor. Assessment of the treatment and time effects is of exploratory interest for the primary outcome in testing for a possible interaction, and both treatment and time will be used as systematic factors, using the baseline value as covariate to reduce random variation and increase power.

For all of the above analyses, results will be expressed as the difference between the group means and 95% confidence intervals with associated *P* values, based on the mixed linear model. All data analyses will be carried out according to the pre-established analysis plan, and will be performed applying SAS software. All descriptive statistics and tests will be reported in accordance with the recommendations of the Enhancing the QUAlity and Transparency Of health Research (EQUATOR) network [48] and the Consolidating Standards of Reporting Trials (CONSORT) statement [49]. In order to evaluate the empirical distributions of the continuous outcomes, visual inspection (of the studentised residuals from the statistical model) will be used to suggest whether the assumption of normality and variance homogeneity is reasonable.

Statistical analysis of the economic variables will be presented as descriptive statistics, and a cost-utility analysis of the economic burden of each specific exercise programme in relation to the EQ-5D will be performed, expressed in quality-adjusted life years (QALYs) gained.

### Interim analysis and early stopping rules

An early stopping rule will be applied when complete rotator cuff ruptures, verified by ultrasonography, occur due to the performance of the exercise programme. If six or more cases of complete rupture occur in the progressive high-load exercise group, and the rate of rupture is 50% more compared with the low-load exercise group, the trial will be stopped.

### Data monitoring

Since adverse events are expected to be minimal and the intervention is not considered a high-risk intervention, no data monitoring committee will be established. The physiotherapists at each centre will be asked to report adverse events to the primary investigator, who will report these to the ethics committee and monitor if the number of adverse events has reached the threshold for the early stopping rule.

### Ethics

All patients will participate on a voluntary basis and will be informed that they can withdraw at any time without this influencing their subsequent treatment. Informed consent will be obtained from all patients. The Regional Scientific Ethics Committee of Southern Denmark approved the trial on the 27 June 2013 (project ID: S-20130071). The trial was registered with the Danish Data Protection Agency and approved on the 30 May 2013 (registration number: 2008-58-0035). The trial is registered at Clinicaltrials.gov (identifier: NCT01984203). The Danish Act concerning Processing of Personal Data (DAPPD) will be followed. The trial will follow the principles of the Declaration of Helsinki [50].

## Discussion

The effect of exercise on the rehabilitation of SIS has been well studied, but the latest systematic reviews focusing on active exercise rehabilitation of SIS consistently report that most studies are of low to moderate quality [8,51-57]. This is primarily due to small sample sizes, inadequate blinding of patients and/or investigators and incomplete intervention descriptions, making it difficult to translate the results into clinical practice. Furthermore, previous studies show a wide range in clinical outcome (from some effect to almost no effect), which is probably the result of mixed populations within the SIS ‘umbrella of pathologies’, and variation in the interventions used [8]. However, collectively there is evidence that active exercise is effective in the rehabilitation of SIS. When evaluating new treatment approaches that already have some evidence of efficacy, it is considered unethical to compare treatment with a placebo or similar. New approaches should at least always be tested against current best practice.

The RoCTEx trial will add new knowledge to the field as described in the following points. Firstly, the focus on patients with rotator cuff tendinopathy will result in a more homogeneous group, giving the possibility to transfer exercise principles (such as progressive high-load exercise) to the shoulder that previously have been shown to have positive results in other body regions. The specific exercises for this trial have been selected on the basis of evidence from Electromyography (EMG) studies, resulting in targeting primarily the m. supraspinatus, which is most often considered the site of tendinopathy. In addition, the trial’s exercises will be focusing on stabilising muscles for the scapulae, thereby providing the foundation for optimal functioning of the glenohumeral joint [58-61]. Secondly, efforts will be made to keep both primary assessors and patients blinded to the intervention group, which has been one of the most frequent criticisms of earlier studies. Thirdly, we will include an active rehabilitation strategy for our control group in order to specify if the effect of the intervention is due specifically to the high-load exercise, or merely to the effect of improved muscle activation of the shoulder muscles. Fourthly, allowing for a subgroup of the included patients having a corticosteroid injection if deemed necessary by the orthopaedic specialist, and subsequently stratifying for this effect, increases the external validity of the results.

Since no gold standard exists for diagnosing rotator cuff tendinopathy, the current inclusion criteria may be questioned. The inherent diagnostic difficulties could result in the inclusion of patients not having rotator cuff tendinopathy, thereby increasing participant variability and thereby make it more difficult to reach a significant result. However, our sample size is calculated on the basis of studies with the same variation in populations, thereby presumably taking this variation into account. Furthermore, ultrasound has shown moderate sensitivity and excellent specificity in diagnosing rotator cuff tendinopathy. The excellent specificity of 0.88 suggests an acceptable probability of not including patients who do not have rotator cuff tendinopathy [62].

The multifactorial pathophysiology of SIS (rotator cuff pathology and degeneration based on decreased vascularity, scapular dyskinesia, instability of the glenohumeral joint or glenohumeral internal rotation deficit) may result in patients not responding positively to a single modality exercise programme. However, as we focus our inclusion on those patients who have signs of rotator cuff tendinopathy, and only distinguish the intervention and control groups by the external load applied, we expect that this will not excessively dilute the results. Furthermore, the trial’s exercise programme will consider the multifactorial origin by including exercises focused on the overall strength, stability and mobility of the scapula-humeral complex.

The progressive high-load exercise programme is optimised in several ways that distinguish it from traditional eccentric exercise programmes. The concentric heavy slow resistance exercises are favourable because of their similarity to daily activities, thereby presumably increasing the likelihood of compliance. Furthermore, eccentric exercises for the rotator cuff muscles may induce a more unsuitable initial position, reducing the subacromial space, with an anticipated increased risk of subacromial impingement. Alternatively, using exercises starting with a concentric phase, and allowing the patients only to do isometric exercises when restricted by pain, will potentially minimise the risk of irritating the subacromial structures.

The objective of the RoCTEx trial is to show whether there is a significant advantage in progressive high-load exercise compared with the more traditional low-load exercise. If this is the case, the description and availability of a standardised progressive high-load exercise programme will help practitioners to treat patients diagnosed with rotator cuff tendinopathy. If no difference occurs between the two groups, practitioners will have the possibility of allowing their patients to choose the exercise modality that best optimises their compliance.

## Trial status

At the time of submission of this study protocol, the trial is ongoing and still recruiting patients. Recruitment began November 2013, and is expected to end Marts 2015.

## Abbreviations

ANCOVA: ANalysis of COVAriance
BOCF: Baseline Observation Carried Forward
CANS: Complaints of the Arm, Neck and/or Shoulder
CONSORT: CONsolidating Standards Of Reporting Trials
DAPPD: Danish Act concerning Processing of Personal Data
DASH: Disabilities of the Arm, Shoulder and Hand questionnaire
EMG: ElectroMyoGraphy
EQUATOR: Enhancing the QUAlity and Transparency Of health Research
HAD: Hospital Anxiety and Depression Scale
ITT: Intention-To-Treat
LLE: Low-Load Exercises
MVC: Maximum isometric Voluntary Contraction
PHLE: Progressive High-Load Exercises
QUALY: QUality-Adjusted Life Years
RM: Repetitions Maximum
RC: Rotator Cuff
RoCTEx: Rotator Cuff Tendinopathy Exercise
SAT: Scapulae Assistance Test
SD: Standard Deviations
SIS: Subacromial Impingement Syndrome
SRT: Scapulae Retraction Test
SOOS: Shoulder injury and Osteoarthritis Outcome Score
VAS: Visual Analogue Scale
